# Interactions between maternal parity and feed additives drive the composition of pig gut microbiomes in the post-weaning period

**DOI:** 10.1186/s40104-024-00993-x

**Published:** 2024-03-03

**Authors:** Kayla Law, Eduardo Rosa Medina Garcia, Chad Hastad, Deborah Murray, Pedro E. Urriola, Andres Gomez

**Affiliations:** 1 Department of Animal Science, University of Minnesota, 1364 Eckles Avenue, Saint Paul, MN 55108 USA; 2 New Fashion Pork, 164 Industrial Parkway, Jackson, MN 56143 USA

**Keywords:** *Aspergillus* prebiotic, Copper, Feed additives, Maternal, Nursery pig microbiome, Parity, Swine microbiome, Trace minerals, Zinc

## Abstract

**Background:**

Nursery pigs undergo stressors in the post-weaning period that result in production and welfare challenges. These challenges disproportionately impact the offspring of primiparous sows compared to those of multiparous counterparts. Little is known regarding potential interactions between parity and feed additives in the post-weaning period and their effects on nursery pig microbiomes. Therefore, the objective of this study was to investigate the effects of maternal parity on sow and offspring microbiomes and the influence of sow parity on pig fecal microbiome and performance in response to a prebiotic post-weaning. At weaning, piglets were allotted into three treatment groups: a standard nursery diet including pharmacological doses of Zn and Cu (Con), a group fed a commercial prebiotic only (Preb) based on an Aspergillus oryzae fermentation extract, and a group fed the same prebiotic plus Zn and Cu (Preb + ZnCu).

**Results:**

Although there were no differences in vaginal microbiome composition between primiparous and multiparous sows, fecal microbiome composition was different (*R*^2^ = 0.02, *P* = 0.03). The fecal microbiomes of primiparous offspring displayed significantly higher bacterial diversity compared to multiparous offspring at d 0 and d 21 postweaning (*P* < 0.01), with differences in community composition observed at d 21 (*R*^2^ = 0.03, *P* = 0.04). When analyzing the effects of maternal parity within each treatment, only the Preb diet triggered significant microbiome distinctions between primiparous and multiparous offspring (d 21: *R*^2^ = 0.13, *P* = 0.01; d 42: *R*^2^ = 0.19, *P* = 0.001). Compositional differences in pig fecal microbiomes between treatments were observed only at d 21 (*R*^2^ = 0.12, *P* = 0.001). Pigs in the Con group gained significantly more weight throughout the nursery period when compared to those in the Preb + ZnCu group.

**Conclusions:**

Nursery pig gut microbiome composition was influenced by supplementation with an *Aspergillus oryzae* fermentation extract, with varying effects on performance when combined with pharmacological levels of Zn and Cu or for offspring of different maternal parity groups. These results indicate that the development of nursery pig gut microbiomes is shaped by maternal parity and potential interactions with the effects of dietary feed additives.

**Supplementary Information:**

The online version contains supplementary material available at 10.1186/s40104-024-00993-x.

## Background

The microbes seeding piglet microbiomes are first acquired from their mother, as well as from the surrounding environmental surfaces [1–3]. Within just the first 24 h of life, piglet microbiomes undergo rapid colonization and taxonomic shifts [4, 5]. This initial wave of pioneer microorganisms is thought to also be driven by the ingestion of sow colostrum immediately post-birth [2, 6]. Pioneer microbes then influence the following successive waves of colonization in the gastrointestinal tract in early life, with long-lasting implications for microbiome community composition, immune development, health, and growth [3, 6–8]. Previous reports regarding piglet microbiome development in early life have observed variations in piglet microbiome acquisition and composition based on rearing conditions, maternal diet, maternal origin, and farm management practices [6, 8–10]. The effect of maternal origin may have the greatest influence on offspring microbiome development, with more compositional similarities observed in related piglets and their mothers [1, 2, 6]. However, whether variations in microbiome development prior to weaning are maintained throughout pigs; lifetimes is unclear [4, 11].

Maternal parity has also been observed to impact the gut microbiomes of gestating sows as well as influence the composition of the gut microbiomes of their offspring in early life [12, 13]. However, the mechanisms driving these observations are currently unknown. The effects of maternal parity on sow and offspring performance have been well documented, with multiparous mothers and offspring displaying greater performance metrics such as increased piglet body weight, increased litter sizes, and greater piglet health status when compared to primiparous mothers and offspring [13]. It is hypothesized that primiparous mothers differ because they have not previously undergone the physiological and hormonal changes associated with pregnancy, and therefore nutrient requirements for gestating sows vary according to parity [14–17]. Additionally, primiparous mothers spend less time in a particular herd or environment than their multiparous counterparts, which may impact their environmental exposures and microbial communities. Some previous reports have proposed that observed differences in offspring development and performance may be linked to differences in colostrum and milk nutrient quality between primiparous and multiparous animals, though these associations and the role of the gut microbiome in performance differences based on parity remain unclear [13, 18, 19].

A myriad of dietary feed additives and supplements have been explored in attempts to bridge observed gaps in performance between primiparous and multiparous mothers and offspring, as well as prevent health challenges in the post-weaning period [7, 20]. In contrast to dietary interventions such as direct-fed microbials (DFMs) and probiotics that rely on the successful assimilation of novel microorganisms into established microbial communities, prebiotic supplementation involves the growth promotion of beneficial microorganisms in the gastrointestinal tract through the provision of fermentable nutrient sources that are otherwise indigestible to the host [21, 22]. Thus, the investigation of prebiotics in nursery diets may provide a more promising approach than other potential dietary interventions aiming to beneficially modulate host microbiomes [21, 22]. Specifically, the use of *Aspergillus oryzae* and its fermentation products as a dietary prebiotic and/or postbiotic is attractive to producers in swine systems because of their effects on intestinal integrity and fermentative microbiome functions [23], and the promotion of potentially beneficial microorganisms with immunomodulatory roles such as species of *Lactobacillus* [24], which are considered indicators of optimal intestinal health [6, 20]. However, reports on the efficacy of a wide range of prebiotics are still highly variable in swine systems [7, 20].

Studies involving prebiotic supplementation in nursery pig diets overwhelmingly lack information and discussions regarding two crucial methodological details, potentially contributing to observed variations in efficacy in swine systems. Firstly, most studies involving nursery pigs and dietary supplements do not account for the potential inclusion of pharmacological levels of zinc (Zn) and copper (Cu) in commercial nursery pig diets. Supplementation of Zn and Cu in excess of nutrient requirement guidelines [25] is a common practice globally to achieve growth promotion and to reduce incidences of post-weaning diarrhea and disease [26, 27], with the exception of the European Union due to recent regulations [28]. However, the exact levels of Zn and Cu supplementation and their relative bioavailability in combination with other supplements are also subject to variation [29, 30]. Therefore, it is unknown whether the inclusion of pharmacological levels of Zn and Cu impacts the effectiveness of prebiotics in terms of microbiome modulation and growth promotion. Secondly, the potential impacts of sow parity on the effectiveness of dietary prebiotics are also largely unexplored. Feeding trials are typically designed to account for maternal parity in their experimental design by balancing treatment groups to have proportionate amounts of offspring from both primiparous and multiparous mothers. However, longitudinal microbiome studies and feeding trials accompanied by microbiome analyses in swine systems often neglect the potential effects of maternal origin and parity in their analyses [6, 11, 31]. The high variability in reported results of different dietary supplements for nursery pig growth and microbiome development [7, 20] may therefore potentially be attributed to these two overlooked factors.

The objectives of this study were to investigate the effect of maternal parity on sow and offspring microbiomes, and the influence of parity on pig gut microbiome composition and performance in response to a post-weaning prebiotic. To this end, we attempted to explore the potential relationships between dietary prebiotics, supplementation of pharmacological levels of Zn and Cu, and maternal parity in the context of nursery pig growth and their microbiomes. Specifically, we first focus on determining whether parity influences sow vaginal, sow gut, and piglet gut microbiomes at weaning. Next, we focus on determining the effects of an *Aspergillus oryzae* prebiotic with and without the inclusion of pharmacological levels of Zn and Cu on nursery pig performance and microbiomes. Lastly, we explore the potential effects of maternal parity on offspring microbiomes throughout the nursery period and attempt to disentangle potential interactions between maternal parity and maternal dietary treatments aimed at improving piglet growth performance.

## Methods

### Experimental design

A total of 96 mixed-parity crossbred sows (*n* = 21 primiparous and *n* = 75 multiparous) from the Freking sow farms operated by New Fashion Pork Inc., Jackson, MN and their offspring (PIC^®^ TR4 × [Fast LW × PIC^®^ L02, Hendersonville, TN]) were enrolled in this study [32]. All research in this study was conducted using animal care and use practices in accordance with the Animal Use for Research and Scientific Purposes Act and Directive 2010/63/EU guidelines and was supervised by an attending veterinarian from New Fashion Pork. At weaning (~ d 21), piglets (*n* = 600) were transported to the Koster Research Nursery facility in Round Lake, MN and allotted into pens using a randomized complete block design on the basis of body weight. Pigs were separated into 30 total (10 pens/treatment) grated-floor pens (4.8 m × 2.4 m) at a rate of 20 pigs per pen. Each pen was equipped with plastic-grate flooring, a one cup drinker, and a 4-hole stainless steel self-feeder (Hog Slat Inc., Newton Grove, NC, USA). Pens were then randomly assigned to one of three dietary treatment groups: a commercial nursery diet including industry-standard pharmacological doses of Zn and Cu (control, Con), a group fed the same diet but with a 0.5% inclusion rate of a commercial prebiotic and no pharmacological doses of Zn and Cu (Preb), and a group fed the same prebiotic (0.5% inclusion) plus the pharmacological levels of Zn and Cu (Preb + ZnCu). Nursery diets were fed in four phases (Additional file 1), with changes between phases managed by pre-established estimates of feed budgets per pig (Phase 1: 2.42 kg, Phase 2: 3.63 kg, Phase 3: 3.63 kg, Phase 4: ad libitum). Pharmacological doses of Zn and Cu were only included in the first two dietary phases for treatments Con and Preb + ZnCu, while prebiotic supplementation occurred only in the first three dietary phases for treatments Preb and Preb + ZnCu. Dietary phase 4 consisted of a common diet for all pens and treatment groups. All diets were formulated to meet NRC recommendations for nursery pigs [25]. The prebiotic consisted of an *Aspergillus oryzae* fermentation extract (Amaferm^®^, BioZyme Inc., St. Joseph, MO, USA). Pharmacological doses of Zn and Cu were included in diets in the form of ZnO (2,880 mg/kg) and tribasic CuCl_2_ (232 mg/kg), as previously described [33]. Pigs had ad libitum access to feed and water throughout the study. Individual pig body weight was recorded at weaning (d 0) and at the end of the nursery period (d 42). Average daily gain (ADG) was calculated for the entire nursery period by dividing total weight gain by the length of the study (42 d). Feed disappearance was recorded weekly by pen by subtracting the weight of the feeder from the amount of feed added in the last week. Average daily feed intake (ADFI) was determined by dividing feed disappearance by the number of pigs in each pen.

### Microbiome sampling and sequence data processing

All fecal samples for microbiome analyses were collected using sterile cotton swabs and collection tubes. Fecal swabs were collected by inserting the tip of the cotton swab just inside the rectum. Vaginal swabs were collected by first wiping the exterior vulva opening with sterile gauze pads soaked in 70% ethanol to remove debris, soaking the cotton swab with sterile PBS, and then inserting the tip of the cotton swab just inside the vulva. Vaginal and fecal swabs were collected from all 96 sows on the d of weaning and piglet separation (d 0) [34]. Fecal swabs were collected from 48 randomly selected pigs (*n* = 16 per dietary treatment group; *n* = 24 primiparous offspring and *n* = 24 multiparous offspring) at weaning (d 0), d 21 post-weaning, and d 42 post-weaning. All samples were immediately placed on dry ice after collection and then stored at −80 °C prior to sample processing.

DNA was extracted from swab samples using Qiagen PowerSoil DNA extraction kits (Qiagen, Hilden, Germany), with negative controls created for each set of extraction kit reagents to account for potential contamination from reagents or other environmental sources. Extracted DNA was sequenced on the MiSeq sequencing platform by targeting the V4 variable region of the 16S rRNA bacterial gene using dual-indexing library preparation and the primers 515F (5´-GTGCCAGCMGCCGCGGTAA-3´) and 806R (5´-GGACTACHVGGGTWTCTAAT-3´) [35]. Raw sequence data was processed to trim primer sequences and quality filter reads, as previously described [9]. Raw sequence data contained an average of 22,859 ± 8,113 forward/reverse reads per sample (range, 103 to 49,278 reads/sample). Processed sequence data had an average of 20,094 ± 7,163 reads (range, 53 to 45,157 reads/sample). Appropriate sequencing coverage across all samples was ensured through the filtering of samples to only include those with a depth of at least 2,000 reads, which resulted in the exclusion of one sow vaginal sample (Additional file 2). Processed sequence data were then assigned amplicon sequence variants (ASVs) using the QIIME2 pipeline [36], along with the DADA2 plug-in [37] and the Greengenes database (v13.8) [38].

### Statistical analysis

All data analyses and statistical analyses were performed using the R statistical interface [39]. Sequence data generated from negative controls were used to screen processed ASV-level sequence data for potential contamination using the prevalence method of the R decontam package [40]. Briefly, the prevalence method identifies contaminants based on the presence of taxa in samples versus their corresponding control samples as well as the relative frequency at which they appear. Identified contaminants were subsequently filtered out of processed sequence data sets. Sequence data were then filtered to account for potential sequencing artifacts by removing ASV’s present at extremely low frequencies (*n* < 5) or in 3 or fewer samples using the R labdsv package [41].

Alpha diversity analyses, beta diversity Bray–Curtis analyses, permutational multivariate analyses of variance (PERMANOVA) models, and analyses of similarities (ANOSIM) were created using the R vegan package [42]. Principal-coordinate analyses (PCoAs) based on Bray–Curtis distances were calculated using the R ape package [43], and visualized and plotted using base R plotting functions. Discriminant taxa were identified using species indicator analyses within the R labdsv package [41]. Indicator species analyses account for both average relative abundances of taxa and how frequently they are identified in a specified group, with a perfect indicator value of 1 indicating that a given taxon is present in all samples of a group and occurs in high abundances compared to another group. Network analyses were created using false discovery rate (FDR)- adjusted and compositionally corrected Spearman correlation matrices calculated by the R package ccrepe [44], and visualized and plotted using Cytoscape [45]. With the exception of network analyses and PCoAs, all other figures were created using the R package ggplot2 [46]. All figures were created using the viridis color scales for colorblind-friendly visualizations [47].

Performance data for nursery pigs was analyzed using a generalized linear mixed model (GLMM) using the R package lme4 [48]. Models for weight gain and ADG considered treatment and maternal parity group as fixed effects, and the random effects of treatment nested within block. The model for ADFI was similar, but with an additional fixed effect of time and the pen as the experimental unit. Statistical significance testing for nonparametric analyses was performed using Wilcoxon tests, Kruskal–Wallis tests, or PERMANOVAs. Statistical significance in figures is denoted with three asterisks when the *P* value is < 0.001, two asterisks when the *P* value is < 0.01, one asterisk when the *P* value is < 0.05, and a cross when the *P* value is < 0.1. Figures with alphabetical superscripts denote statistically significant differences ( *P* < 0.05) where letters differ.

## Results

**Fig. 1:**
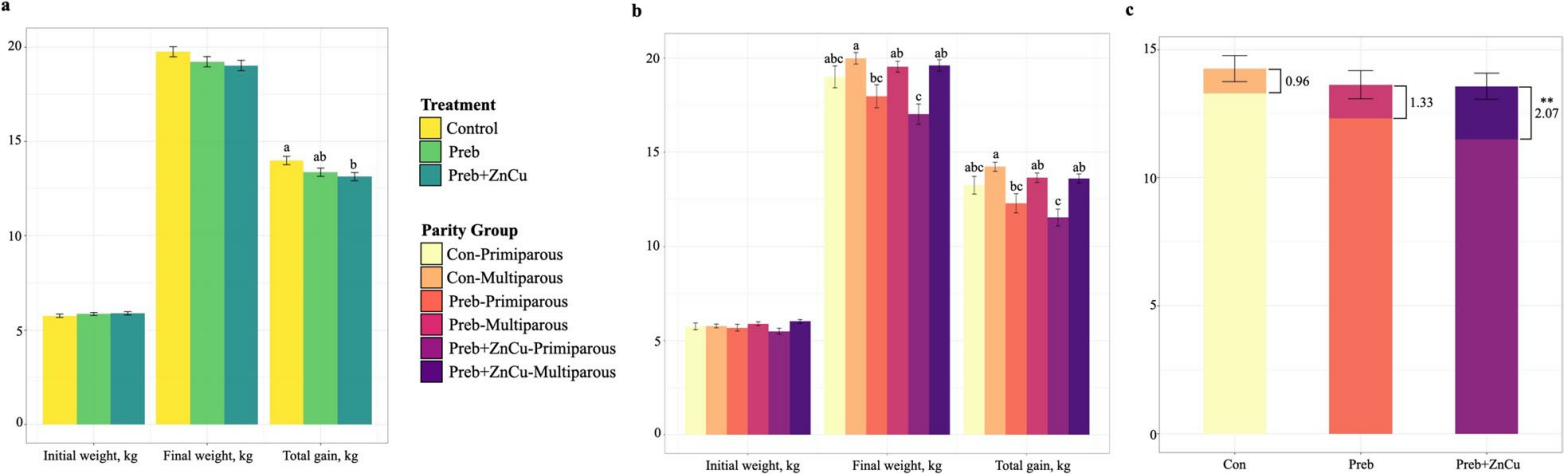
Growth performance results. Average individual nursery pig performance data is displayed by (**a**) dietary treatment group and (**b**) dietary treatment groups split by maternal parity group. Differing letter subscripts denote significant (*P* < 0.05) differences between groups, within each panel. Error bars represent standard error. Total gain of dietary treatment groups split by maternal parity group is displayed again in (**c**) where gaps in performance between maternal parity groups are denoted by stacked bars for each treatment group. Primiparous total gain is represented by the bottom color of each bar, with the top color representing the difference in performance between primiparous and multiparous offspring. Numerical values denote estimated differences (least square means) in means between parity groups, and error bars represent standard error for estimated differences

While multiparous sows had more pre-weaning mortalities compared to primiparous pigs, total liveborn piglets did not differ between primiparous and multiparous sows (Additional file 3). Individual pig weight from d 0 and d 42 post-weaning was analyzed by dietary treatment alone, as well as by maternal parity group within each treatment. No significant interactions between dietary treatment and maternal parity group were observed for growth performance models. Nursery pigs belonging to the Con group had the greatest total weight gain throughout the nursery period, but statistically significant differences were only observed between the Con and Preb + ZnCu groups (Fig. 1a). In general, pigs born to multiparous mothers displayed numerically higher final weight at the end of the nursery period and greater overall weight gain when compared to offspring of primiparous mothers. Within dietary treatment groups, statistically significant differences in performance metrics between maternal parity groups were observed for pigs receiving the Preb + ZnCu diet (Fig. 1b). Pigs in the Con group displayed the smallest gap in performance between maternal parity groups (Fig. 1b and c). In contrast, the largest performance gap between the offspring of primiparous and multiparous mothers was observed for pigs receiving the Preb + ZnCu diet (Fig. 1b and c). While overall nursery ADG and ADFI did not differ among treatments, pigs in group Preb had decreased ADFI in weeks 2–4 (Additional file 4).

### Maternal parity influences the sow gut microbiome at weaning, but has limited influence on sow vaginal microbiomes

**Fig. 2:**
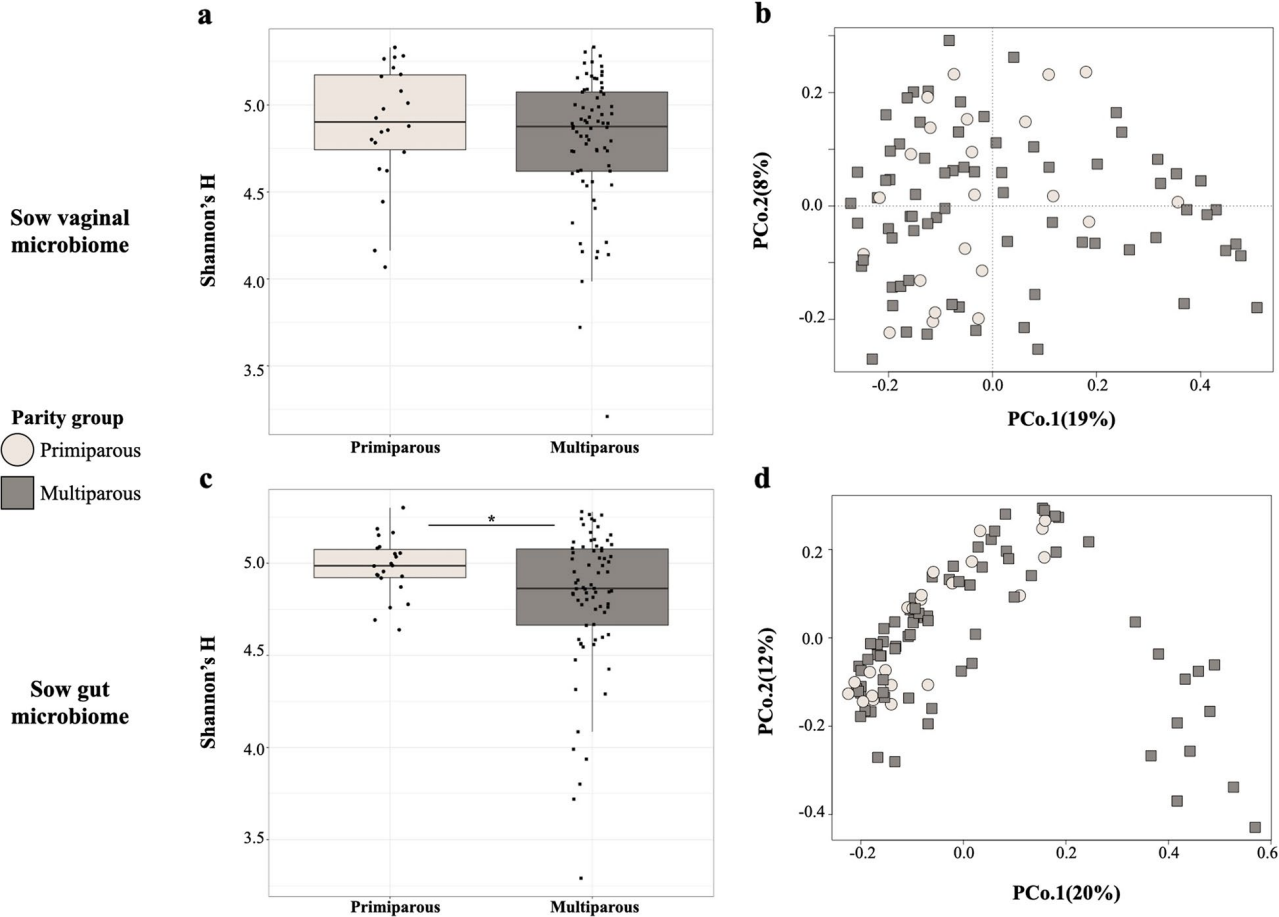
Diversity and composition of sow microbiomes from different parity groups. Bacterial diversity (Shannon’s *H*) and microbiome composition based on parity group is displayed for sow vaginal samples (**a**, **b**) and sow fecal samples (**c**, **d**) at weaning (d 0). Each point or shape represents an individual sample. Circles denote the primiparous maternal parity group, while squares represent the multiparous maternal parity group

No differences were observed in sow vaginal microbiome diversity (Shannon’s *H*, *P* > 0.05; Fig. 2a) or composition at weaning (d 0) based on parity (Bray–Curtis PERMANOVA, F-model = 1.31, ${R}^{2}$ = 0.014, *P* = 0.1; Fig. 2b). However, primiparous sow fecal microbiomes harbored greater microbial diversity (Shannon’s *H*, *P* < 0.05; Fig. 2c) and significantly different community composition (Bray–Curtis PERMANOVA, F-model = 1.90, ${R}^{2}$ = 0.02, *P* = 0.037; Fig. 2d) when compared to the gut microbiomes of multiparous sows.

ASV-level sequence data were collapsed to generate genus-level taxa prior to identification of discriminant taxa. Discriminant genera between parity groups were identified as those with indicator values of at least 0.5. An indicator value of 1.0 indicates that the genus is present in high abundances in all samples of a group, while absent in all samples of the other group(s). The vaginal microbiomes of primiparous sows were characterized by significantly higher relative abundances of the genera *Dorea*, *Megasphaera*, and *Mogibacterium* when compared to the vaginal microbiomes of multiparous sows (Additional file 5). At gut level, the genera *Lachnospira*, *Megasphaera*, and *Anaerovibrio* were enriched in primiparous sows, while *Peptococcus*, *Peptoniphilus*, *Porphyromonas*, *Mobiluncus*, and *Clostridium* characterized multiparous sows (Additional file 6).

### Maternal parity influences nursery pig gut microbiomes at weaning, with diminishing effects over time

**Fig. 3:**
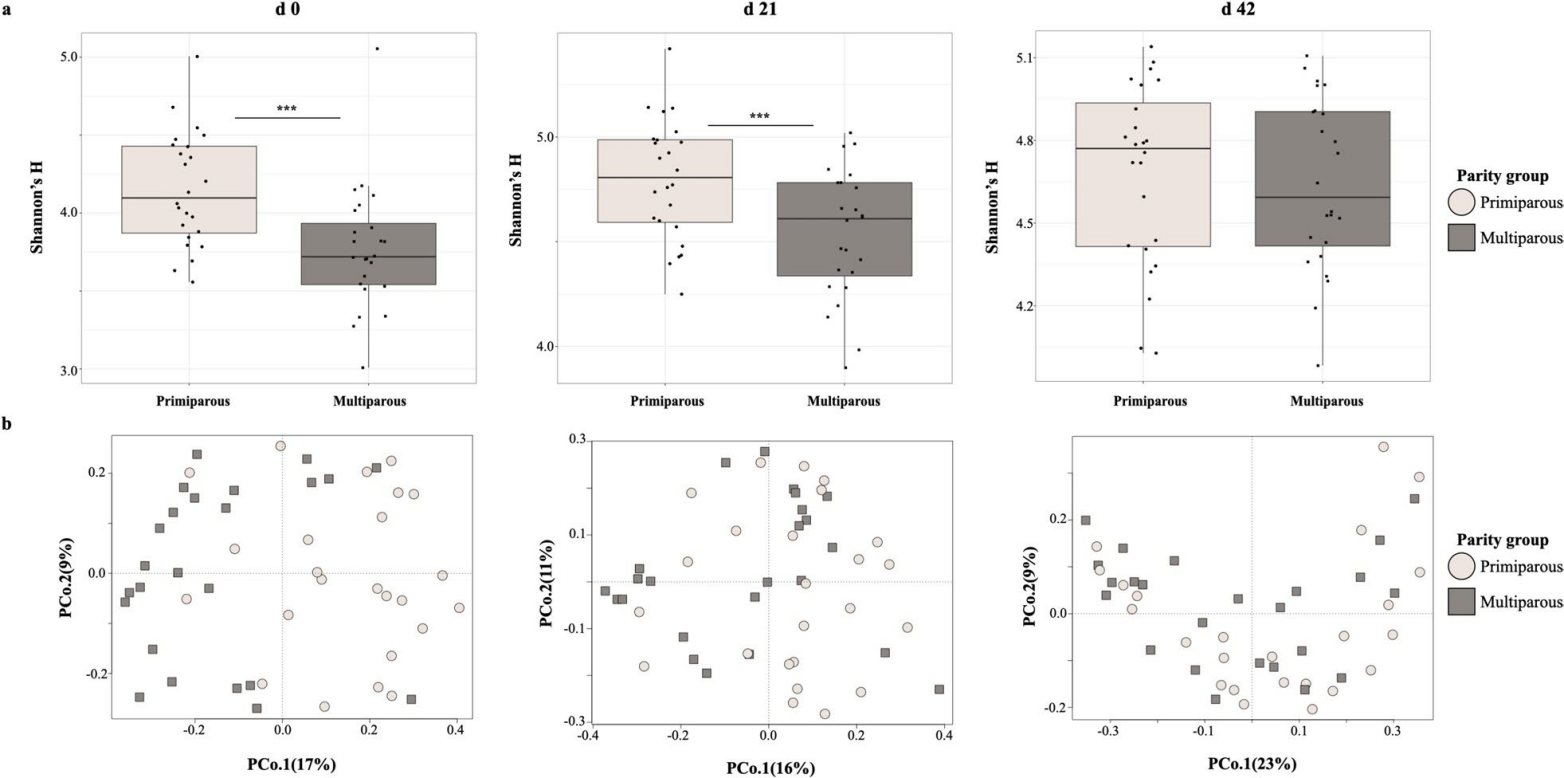
Diversity and composition of nursery pig microbiomes from different parity groups. Nursery pig gut microbiome microbial diversity (**a**) and composition (**b**) separated by maternal parity group, from d 0 to d 42. Each point or shape represents an individual sample. Circles denote the primiparous maternal parity group, while squares represent the multiparous maternal parity group

The gut microbiomes of offspring born to primiparous mothers displayed significantly higher microbial diversity when compared to multiparous offspring at d 0 post-weaning (Shannon’s *H*, *P* < 0.001) (Fig. 3a). This trend continued at d 21 post-weaning (Shannon’s *H*, *P* < 0.01), but was not observed by d 42 (Shannon’s *H*, *P* > 0.05) (Fig. 3a). At weaning (d 0), pig gut microbiome composition was distinct between primiparous and multiparous offspring (Bray–Curtis PERMANOVA, F-model = 4.26, ${R}^{2}$ = 0.08, *P* = 0.001; ANOSIM’S *R* = 0.18, *P* = 0.001) (Fig. 3b). Several genera were identified as discriminant taxa between primiparous and multiparous offspring at weaning (d 0) (Table 1). These patterns in microbiome composition were still evident, albeit with diminished effect sizes, at d 21 post-weaning (Bray–Curtis PERMANOVA, F-model = 1.55, ${R}^{2}$ = 0.03, *P* = 0.04; ANOSIM’S *R* = 0.06, *P* = 0.002) (Fig. 3b). By d 42 post-weaning, the effect of maternal parity on nursery pig gut microbiome composition was not as clearly defined (Bray–Curtis PERMANOVA, F-model = 1.05, ${R}^{2}$ = 0.02, *P* = 0.3; ANOSIM’S *R* = 0.03, *P* = 0.048) (Fig. 3b).

**Table 1:** Discriminant genera between parity groups for pig gut microbiomes at weaning (d 0)

**Genus**^1^	**Group**^2^	**Indicator value**	**Average primiparous relative abundance for P, %**	**Average multiparous relative abundance for M, %**	***P*-value**^3^
o__Coriobacteriales.f__Coriobacteriaceae.g__	M	0.86	0.33	2.05	< 0.001
o__Actinomycetales.f__Actinomycetaceae.g__*Actinomyces*	M	0.83	0.08	1.34	< 0.001
o__Bacteroidales.f__Bacteroidaceae.g__*Bacteroides*	M	0.65	6.63	12.41	< 0.01
o__Enterobacteriales.f__Enterobacteriaceae.__	M	0.64	4.42	7.80	< 0.01
o__Methanobacteriales.f__Methanobacteriaceae.g__*Methanobrevibacter*	M	0.62	2.11	3.50	< 0.01
o__Erysipelotrichales.f__Erysipelotrichaceae.g__*Anaerorhabdus*	M	0.62	0.002	0.19	< 0.001
o__Clostridiales.f__Clostridiaceae.g__*Clostridium*	M	0.61	1.16	2.30	< 0.05
o__Erysipelotrichales.f__Erysipelotrichaceae.g__*Clostridium*	M	0.57	0.05	0.32	< 0.01
o__Burkholderiales.f__Alcaligenaceae.g__*Sutterella*	P	0.78	1.03	0.24	< 0.01
o__Bacteroidales.f__S24.7.g__	P	0.77	3.28	1.00	< 0.001
o__Clostridiales.f__Ruminococcaceae.g__	P	0.77	3.35	1.03	< 0.001
o__WCHB1.41.f__RFP12.g__	P	0.76	0.57	0.08	< 0.001
o__Bacteroidales.f__Prevotellaceae.g__*Prevotella*	P	0.75	8.68	2.84	< 0.001
o__Clostridiales.f__Ruminococcaceae.g__*Oscillospira*	P	0.73	3.88	1.40	< 0.001
o__Clostridiales.f__Veillonellaceae.g__*Anaerovibrio*	P	0.7	0.84	0.06	< 0.001
o__Sphaerochaetales.f__Sphaerochaetaceae.g__*Sphaerochaeta*	P	0.68	0.83	0.28	< 0.01
o__Clostridiales.f__Ruminococcaceae.g__*Faecalibacterium*	P	0.65	0.56	0.08	< 0.001
o__Bacteroidales.f__.Paraprevotellaceae..g__	P	0.65	0.33	0.07	< 0.001
c__Clostridia.o__Clostridiales.__.__	P	0.63	0.85	0.38	< 0.01
c__Mollicutes.o__RF39.f__.g__	P	0.63	0.29	0.07	< 0.001
c__Bacteroidia.o__Bacteroidales.f__.g__	P	0.63	1.31	0.78	< 0.01
o__E2.f__.Methanomassiliicoccaceae..g__*vadinCA11*	P	0.62	0.42	0.15	< 0.05
o__Clostridiales.f__Lachnospiraceae.g__*Blautia*	P	0.6	0.52	0.17	< 0.01
o__Campylobacterales.f__Helicobacteraceae.g__*Flexispira*	P	0.56	3.23	0.59	< 0.01
o__Clostridiales.f__.Mogibacteriaceae..g__*Mogibacterium*	P	0.55	0.38	0.14	< 0.05
c__.Lentisphaeria..o__Z20.f__R4.45B.g__	P	0.54	0.15	0.05	< 0.01
o__Clostridiales.f__Lachnospiraceae.g__*Coprococcus*	P	0.53	0.14	0.07	< 0.01
o__Victivallales.f__Victivallaceae.g__	P	0.51	0.14	0.06	< 0.05

^1^Listed taxa were selected on the basis of having indicator value scores of at least 0.5, and significant differences (Wilcoxon test, *P* < 0.05) in average relative abundances between parity groups. Taxa are sorted by parity group and then by indicator value. Listed taxa are identified to the genus (g) level, or represent unidentified genera within the listed family (f), order (o), or class (c), where applicable

^2^Group designations of P or M represent the parity group (Primiparous or Multiparous) the listed taxa is enriched in

^3^*P*-values represent One-tailed Wilcoxon tests between parity groups

### Nursery pig gut microbiome diversity was not affected by prebiotic and Zn-Cu supplementation, and microbiome composition was minimally affected in a time-dependent manner

**Fig. 4:**
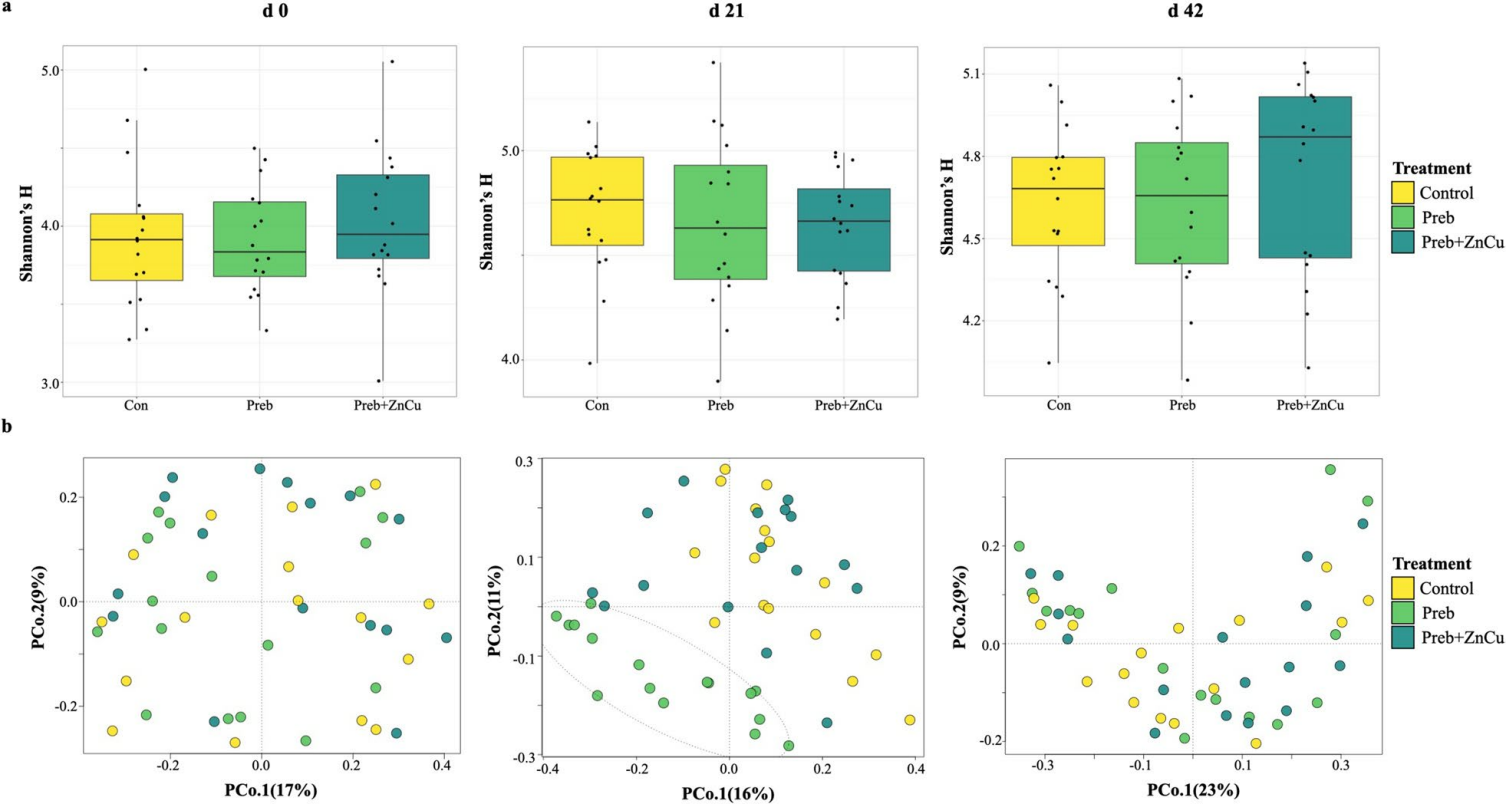
Diversity and composition of nursery pig microbiomes from different dietary treatment groups. Nursery pig gut microbiome microbial diversity (**a**) and composition (**b**) separated by dietary treatment group, from d 0 to d 42. Each point or shape represents an individual sample. The dotted ellipse denotes a 95% confidence interval for treatment Preb at d 21 post-weaning

No significant differences in microbial alpha diversity among dietary treatments were observed in nursery pig gut microbiomes from d 0 to d 42 post-weaning (Shannon’s *H*, Kruskal–Wallis *P* > 0.05; Fig. 4a). At weaning, no significant differences were observed in pig gut microbiome composition among dietary treatments (Bray–Curtis PERMANOVA, F-model = 0.93, ${R}^{2}$ = 0.04, *P* = 0.55) (Fig. 4b). But at d 21 post-weaning, nursery pig gut microbiome composition was distinct and separated according to dietary treatment (Bray–Curtis PERMANOVA, F-model = 2.91, ${R}^{2}$ = 0.11, *P* = 0.001; ANOSIM’S *R* = 0.13, *P* = 0.001) (Fig. 4b), with the pigs receiving the prebiotic alone (Preb) driving most of these differences. In fact, pigs in group Preb were particularly distinguished in comparison to the other two dietary treatments (Fig. 4b, middle panel), showing significantly higher abundances of the genera *Dialister*, *Acidaminococcus*, *Megasphaera*, *Lactobacillus*, and *Oribacterium* (Table 2). However, these observed distinctions were not evident by d 42 post-weaning (Bray–Curtis PERMANOVA, F-model = 1.96, ${R}^{2}$ = 0.08, *P* = 0.7) (Fig. 4b). At d 42 post-weaning, the only differentially abundant genera among treatment groups were the enrichment of the genus *SMB53* within the Clostridiaceae family for the control treatment (Con), and the enrichment of the genus *p.75.a5* within the family Erysipelotrichaeceae for pigs receiving the Prebiotic treatment including Zn and Cu (Preb + ZnCu) (Table 2).

**Table 2:** Discriminant genera among treatments for pig gut microbiomes at d 21 and 42

**Time-point**	**Genus**^1^	**Treat-ment**^2^	**Indicator value**	**Average relative abundance for Con, %**	**Average relative abundance for Preb, %**	**Average relative abundance for Preb + ZnCu, %**	***P*-value**^3^
d 21	o__Clostridiales.f__Clostridiaceae.g__*Clostridium*	Con	0.54	${0.42}^{a}$	${0.05}^{b}$	${0.26}^{a}$	< 0.05
d 21	o__Clostridiales.f__Clostridiaceae.g__	Con	0.53	${4.32}^{a}$	${0.52}^{b}$	${3.33}^{a}$	< 0.05
d 21	o__Clostridiales.f__Clostridiaceae.g__*SMB53*	Con	0.51	${1.26}^{a}$	${0.15}^{b}$	${1.04}^{a}$	< 0.05
d 21	o__Clostridiales.f__Veillonellaceae.g__*Dialister*	Preb	0.74	${0.08}^{a}$	${0.95}^{b}$	${0.25}^{a}$	< 0.05
d 21	o__Pirellulales.f__Pirellulaceae.g__	Preb	0.69	${0.09}^{a}$	${0.53}^{b}$	${0.01}^{a}$	< 0.05
d 21	o__Bacteroidales.f__p.2534.18B5.g__	Preb	0.66	${0.04}^{a}$	${0.20}^{b}$	${0.03}^{a}$	< 0.05
d 21	o__Clostridiales.f__Veillonellaceae.g__*Acidaminococcus*	Preb	0.61	${0.26}^{a}$	${1.27}^{b}$	${0.56}^{ab}$	< 0.05
d 21	o__Clostridiales.f__Veillonellaceae.g__*Megasphaera*	Preb	0.59	${0.75}^{a}$	${4.47}^{b}$	${2.35}^{ab}$	< 0.05
d 21	p__Cyanobacteria.c__4C0d.2.o__YS2.f__.g__	Preb	0.59	${0.38}^{ab}$	${0.96}^{a}$	${0.19}^{b}$	< 0.05
d 21	o__Clostridiales.f__Christensenellaceae.g__	Preb	0.58	${0.79}^{a}$	${1.63}^{b}$	${0.42}^{a}$	< 0.05
d 21	o__Lactobacillales.f__Lactobacillaceae.g__*Lactobacillus*	Preb	0.57	${3.11}^{a}$	${11.0}^{b}$	${5.04}^{a}$	< 0.05
d 21	o__Clostridiales.f__Lachnospiraceae.g__*Oribacterium*	Preb	0.55	${0.13}^{a}$	${0.40}^{b}$	${0.20}^{a}$	< 0.05
d 21	o__Clostridiales.f__Veillonellaceae.__	Preb	0.55	${0.06}^{a}$	${0.74}^{b}$	${0.30}^{a}$	< 0.05
d 21	o__Clostridiales.f__Veillonellaceae.g__*Mitsuokella*	Preb	0.53	${0.42}^{a}$	${1.37}^{b}$	${0.81}^{ab}$	< 0.05
d 42	o__Clostridiales.f__Clostridiaceae.g__*SMB53*	Con	0.46	${1.24}^{a}$	${0.64}^{b}$	${0.82}^{ab}$	0.07
d 42	o__Erysipelotrichales.f__Erysipelotrichaceae.g__*p.75.a5*	Preb + ZnCu	0.45	${0.04}^{a}$	${0.08}^{ab}$	${0.15}^{b}$	0.09

^1^Listed taxa were selected on the basis of having indicator value scores of at least 0.4, and significant differences in average relative abundances between parity groups. Taxa are sorted by treatment group and then by indicator value. Listed taxa are identified to the genus (g) level, or represent unidentified genera within the listed family (f), order (o), class (c), or phylum (p), where applicable

^2^Treatment designations represent the dietary treatment group the listed taxa is enriched in

^3^*P*-values represent Kruskal Wallis tests among treatment groups

^*a,b*^Differing letter superscripts denote significant differences among groups (*P* < 0.05)

From d 0 to 21, no significant interactions between maternal parity and treatment affecting the composition of nursery pig gut microbiomes were observed (d 0: Bray–Curtis PERMANOVA, F-model = 1.09, ${R}^{2}$ = 0.04, *P* = 0.3; d 21: Bray–Curtis PERMANOVA, F-model = 1.06, ${R}^{2}$ = 0.04, *P* = 0.3). However, the interaction between maternal parity and treatment had a significant effect on gut microbiome composition at d 42 post-weaning (Bray–Curtis PERMANOVA, F-model = 1.96, ${R}^{2}$ = 0.08, *P* = 0.006). When pigs were grouped according to maternal parity, discriminant taxa analysis for dietary treatment groups revealed differences in the abundances of several genera (Additional files 7 and 8). However, the genera identified as discriminant for each dietary treatment group differed between parity groups.

### The effects of dietary feed additives on nursery pig microbiomes and performance are disparate between maternal parity groups

**Fig. 5:**
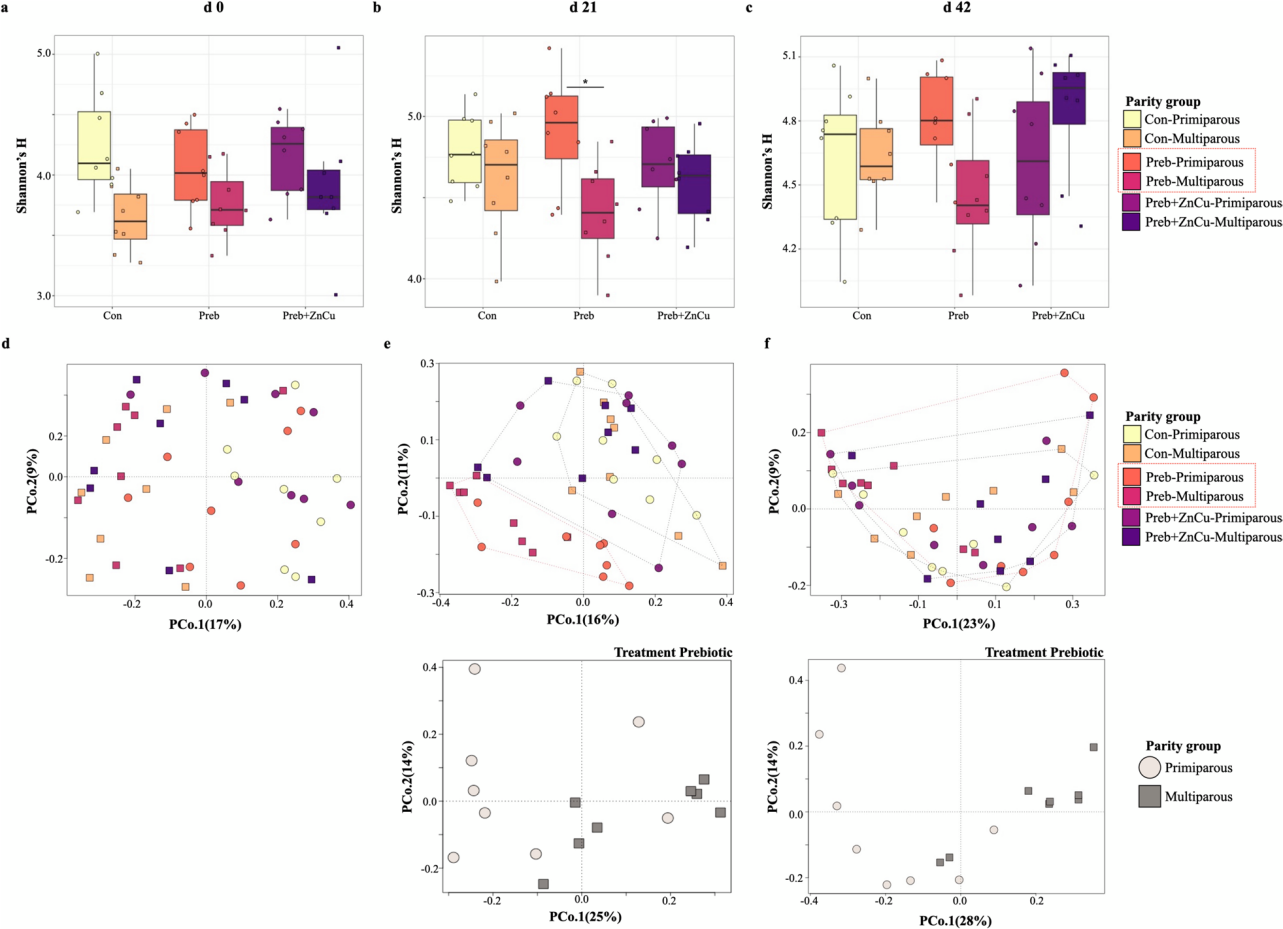
Diversity and composition of nursery pig microbiomes according to both parity and treatment groups. Nursery pig gut microbiome microbial diversity (**a–c**) and composition (**d–f**) separated by both dietary treatment and maternal parity group, from d 0 to d 42. PCoAs displayed under panels (**e**) and (**f**) represent comparisons between maternal parity groups for just treatment Preb. Each point or shape represents an individual sample. Circles denote the primiparous maternal parity group, while squares represent the multiparous maternal parity group. Dotted lines denote dietary treatment groups

The potential interactions between maternal parity and dietary treatment groups were explored further in subsequent analyses. Dietary treatment groups were separated according to maternal parity group to create 6 groups: Con-Primiparous, Con-Multiparous, Preb-Primiparous, Preb-Multiparous, Preb + ZnCu-Primiparous, and Preb + ZnCu-Multiparous. Within dietary treatments, the observed trend for higher microbial diversity in the gut microbiomes of offspring born to primiparous mothers shown in Fig. 3a was mostly maintained, although statistical significance (*P* < 0.05) was only observed at d 21 for pigs in group Preb (Fig. 5a–c). The exception to this observed trend was treatment Preb + ZnCu at d 42 post-weaning, which showed a non-significant numerical tendency for increased microbial diversity in offspring born to multiparous mothers (Fig. 5c). Nursery pig gut microbiome composition displayed distinct differences between parity groups within each dietary treatment group, from weaning to the end of the nursery period (Bray–Curtis PERMANOVA, F-model = 1.36 to 1.95, ${R}^{2}$ = 0.14 to 0.19, *P* = 0.001 to 0.025) (Fig. 5d–f). For pigs in treatment Preb, significant differences in microbiome composition based on maternal parity group were observed at both d 21 post-weaning (Bray–Curtis PERMANOVA, F-model = 2.08, ${R}^{2}$ = 0.13, *P* = 0.013) and d 42 post-weaning (Bray–Curtis PERMANOVA, F-model = 3.33, ${R}^{2}$ = 0.19, *P* = 0.002) (Fig. 5d–f).

### Co-abundance networks of nursery pig gut microbiomes reveal underlying differences in microbiome structure among treatment groups

**Fig. 6:**
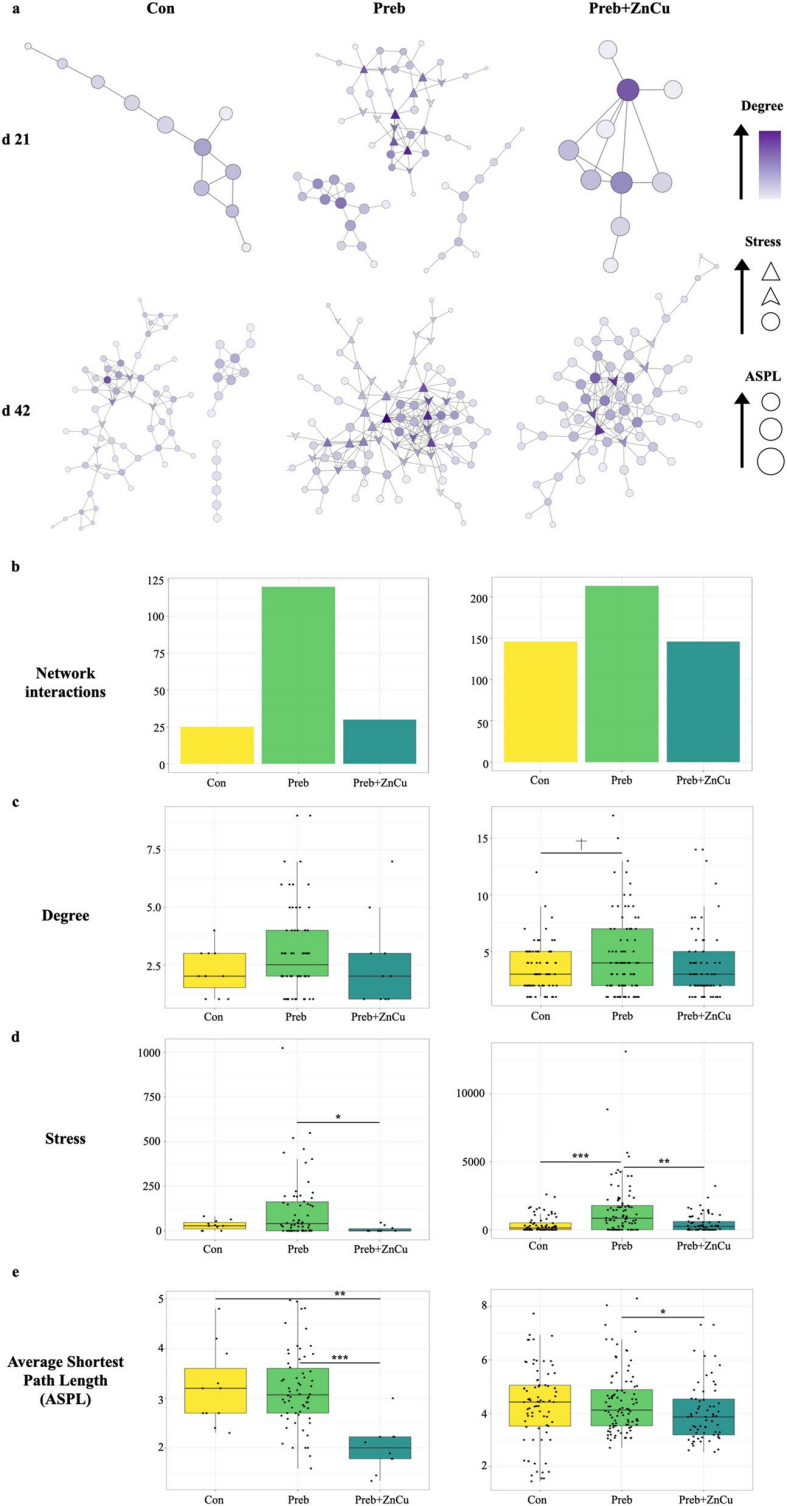
Network analyses of pig microbiomes based on dietary treatment group. (**a**) Network analyses constructed from coabundance matrices are displayed for each dietary treatment group at d 21 and d 42 post-weaning. Each node represents one taxon at the ASV level, with darker shading corresponding to higher degree. Larger node sizes represent decreased amounts of ASPL, and node shapes represent relative levels of stress. Edges represent the undirected interaction or correlation between two nodes. Boxplots display network attributes of networks constructed for nursery pig gut microbiomes for each dietary treatment d 21 and d 42 post-weaning. Network attributes include (**b**) number of significant interactions (*R* > 0.5 and FDR-adjusted *P*-values < 0.05), (**c**) degree, (**d**) stress, and (**e**) Average Shortest Path Length (ASPL). Plots in the left column indicate networks or network attributes at d 21 post-weaning, and plots in the right column indicate the same at d 42 post-weaning throughout the figure

To further explore these results and the potential microbiome characteristics driving observed performance differences among treatment groups, networks modeling microbiome community structure were created for each treatment group at each time-point (Fig. 6a). Networks were created using compositionally-corrected co-abundance matrices between microbes at the ASV level, and were visualized in Cytoscape along with calculations of multiple network topology traits. Each node represents a single taxon at the ASV level. Networks were constructed using significant spearman coefficient correlation values (Rho > 0.5 and FDR-adjusted *P*-values < 0.05), and curated to only include nodes with at least 5 correlations or edges. The number of interactions between taxa identified as significant (q < 0.05 and Rho > 0.5) increased from d 21 to d 42 post-weaning. The number of significant interactions that were identified at each time-point were numerically highest for treatment Preb, which had 120 interactions at d 21 post-weaning and 213 interactions at d 42 post-weaning (Fig. 6a and b). In comparison, networks created for treatments Con and Preb + ZnCu at d 21 post-weaning contained 25 and 30 significant interactions, respectively and at d 42 both contained 146 significant interactions (Fig. 6b). The network traits with the most distinct patterns among treatment groups were degree, stress, and Average Shortest Path Length (ASPL). Degree references the average number of connections a node or taxa has, while network stress is a measure of centrality that quantifies the number of shortest paths flowing through a node. Lower levels of ASPL are associated with increased network connectivity and increased rates of information flow throughout the network.

In accordance with findings regarding a higher number of interactions for the network visualization of the gut microbiome of pigs belonging to treatment Preb, network degree was also numerically greatest for this treatment group. Specifically, degree was numerically higher in the gut microbiomes of pigs in treatment Preb versus Con at d 21 post-weaning, and displayed a tendency (Bonferroni-corrected pairwise Wilcoxon test, *P* = 0.08) for this same pattern at d 42 post-weaning (Fig. 6c). The top three taxa with the highest degree in treatment Preb’s network at d 21 post-weaning were several unidentified species within the genera *Prevotella* (Degree = 9, 7), as well as an unidentified species within the family Coriobacteriaceae (Degree = 9). At d 42 post-weaning, these taxa included unidentified species in the genera *Acidaminococcus* (Degree = 17), *Blautia* (Degree = 15), and *Prevotella copri* (Degree = 15). The network created for treatment Preb was also notable because of numerically greater network stress overall, and due to the observation that stress greatly increased throughout the nursery period in comparison to the other dietary treatment groups (Fig. 6d). Network stress was significantly greater in the microbiomes of pigs belonging to treatment Preb compared to Preb + ZnCu at d 21 post-weaning (Kruskal–Wallis, *P* < 0.05) and compared to both treatments Con and Preb + ZnCu at d 42 post-weaning (Kruskal–Wallis, *P* < 0.05) (Fig. 6d). The highest network stress at d 21 post-weaning was observed in an unidentified species within the family Coriobacteriaceae (Stress = 1,024), as well as unidentified species in the genera *Prevotella* (Stress = 548) and *Megasphaera* (Stress = 520). These taxa shifted to include unidentified species in the genera *Acidaminococcus* (Stress = 13, 118), *Oribacterium* (Stress = 8,860), and *Roseburia* (Stress = 5,662) by d 42 post-weaning. ASPL values were the lowest for the Preb + ZnCu network at d 21 post-weaning (Kruskal–Wallis, *P* < 0.05), but were only significantly decreased in comparison to treatment Preb at d 42 post-weaning (Bonferroni-corrected pairwise Wilcoxon test, *P* < 0.05) (Fig. 6e).

## Discussion

Our results indicate that sow and offspring microbiomes in the post-weaning period are shaped by maternal parity, and provide evidence supporting an association between maternal parity and how nursery pig microbiome and performance respond to dietary interventions in the post-weaning period. The gut microbiomes of primiparous mothers and their offspring were significantly more diverse and compositionally distinct compared to their multiparous counterparts. Also, the gut microbiomes of pigs receiving dietary supplementation consisting of an *Aspergillus oryzae*-based prebiotic underwent marked compositional shifts and fluctuations in the abundances of several taxa, though these distinctions were not observed for pigs receiving diets supplemented with both the prebiotic and pharmacological doses of Zn and Cu. Dietary supplementation with an *Aspergillus oryzae* prebiotic exacerbated observed differences in microbiome composition between offspring of primiparous or multiparous mothers, highlighting maternal parity as a potential driver of variation in effectiveness of dietary feed additives in the post-weaning period.

### Maternal parity influences sow and offspring gut microbiome compositions at weaning

Sow and piglet gut microbiomes were significantly affected by maternal parity at weaning (d 0), in terms of both microbial diversity and composition (Fig. 2c, d and 3a, b). A previous longitudinal study following mixed-parity sows throughout gestation observed that distinctions between primiparous and multiparous sows’ gut microbiome compositions increased in strength from early to late gestation [12]. Thus, the relatively small effect size of maternal parity on sow gut microbiome composition observed in this study may be associated with the collection of sow samples after the gestation period. Furthermore, in previous studies, it was observed that only one prior pregnancy was sufficient to cause large shifts in microbiome community composition compared to primiparous animals [12], indicating that sampling at weaning in this study may have been too late to effectively characterize differences in community composition based on maternal parity. Contrary to previous literature regarding the effects of parity on vaginal microbiome composition [49–51], no differences were observed in our results between maternal parity groups **(**Fig. 2a and b). Therefore, the effects of maternal parity on sow vaginal microbiomes as it pertains to diverse experimental conditions and environments have yet to be clearly defined.

Notably, several parallels between sow and offspring microbiomes were observed regarding the abundances of specific genera according to maternal parity (Additional files 5 and 6, Table 1). *While interest in the seeding and assembly of piglet microbiomes in early life has grown in recent years, prior research has not yet identified distinct connections between the abundances of specific taxa in maternal microbiomes and the abundances of those taxa in offspring microbiomes in the post-weaning period* [2, 3]. Although multiple studies have observed a significant effect of litter or mother on offspring microbiome composition [2, 12], the exact mechanisms driving offspring microbiome acquisition and development remain unclear at this time, and evidence of direct transferal of specific bacterial taxa to offspring is currently inconclusive. Interestingly, differences in offspring gut microbiome composition based on maternal parity were evident throughout the nursery period despite the imposition of dietary treatments, albeit at a diminished effect size over time (Fig. 3b). Microbial diversity increased over time for all pigs (Fig. 3a and 5a–c), in keeping with previous reports of increasing microbial diversity with age [4, 11, 52]. Greater microbial diversity in the gut microbiomes of pigs born to primiparous mothers was observed from d 0–21 post-weaning, but no differences in microbial diversity based on maternal parity group were observable by d 42 post-weaning (Fig. 3a). The lack of observable differences in microbial diversity at the end of the nursery period may be attributed to the overall diversification of pig microbiomes with increasing age, as well as their convergence to a common stage-associated microbiome [11, 31].

### Dietary supplementation with an *Aspergillus oryzae* prebiotic was associated with enduring structural remodeling of microbial communities

Nursery pig gut microbiome composition was distinct among dietary treatment groups at specifically d 21 post-weaning (Fig. 4b), with a loss of observable differences among treatments by d 42 post-weaning in parallel to the transition to a common diet. Microbial diversity was not affected by prebiotic supplementation or the inclusion of pharmacological doses of Zn and Cu (Fig. 4a), contrary to previous reports of decreased microbial diversity after heavy metal supplementation [53]. Pigs belonging to treatment Preb formed an isolated cluster at d 21 post-weaning (Fig. 4b), with no observable similarities in microbiome composition compared with treatment Preb + ZnCu, even though these diets included the same dietary prebiotic. Of the 14 genera that were identified as discriminant taxa among treatment groups at d 21 post-weaning (Table 2), 10 of these genera were unique to treatment Preb. Prebiotic supplementation without the inclusion of pharmacological doses of Zn and Cu (Preb) was associated with increased abundances of the genus *Lactobacillus* at d 21 post-weaning ​​(Table 2), which has previously been linked to increased post-weaning growth rates [54]. However, the pigs receiving the prebiotic alone (Preb) did not display increased or distinct growth performance when compared to the other dietary treatment groups receiving pharmacological doses of Zn and Cu (Fig. 1a and b). These observations can most likely be attributed to the known growth promoting effects of pharmacological doses of heavy metals, as well as observed decreased feed intake in weeks 2–4 post-weaning for pigs in the Preb group (Additional file 4).

Pharmacological levels of Zn and Cu are often included in nursery pig diets for their antimicrobial and growth promoting purposes [26, 27, 29, 55], although this practice is now banned in the European Union due to environmental concerns [30, 56]. Our results regarding increased total gain throughout the nursery period for the Con diet containing pharmacological levels of Zn and Cu (Fig. 1a) support previous reports linking heavy metal supplementation with increased ADG and overall weight gain [29, 55, 57]. The inclusion of pharmacological doses of Zn and Cu in nursery diets is also associated with distinct shifts in microbiome composition [53, 58, 59], although reports of differential abundances of specific taxa are highly variable in accordance with varying experimental conditions [60]. Observed overlaps in microbiome composition between treatments Con and Preb + ZnCu (Fig. 4b) may be attributed to the mutual inclusion of pharmacological doses of Zn and Cu in these diets. However, nursery pig growth performance was significantly different between these treatment groups (Fig. 1a). Additionally, the gap in performance between primiparous and multiparous offspring was greatest for treatment Preb + ZnCu (Fig. 1c). Because high levels of Zn can interact with other components present in swine diets [29, 30], it can be hypothesized that decreased gain for pigs in the Preb + ZnCu group compared to the Con group were associated with an unknown antagonistic combinatory effect of the prebiotic and the pharmacological doses of Zn and Cu together.

The exploration of the microbiomes of dietary treatment groups through the creation of network analyses from co-abundance matrices revealed underlying differences in the assembly and structure of their respective gut microbiome communities, regardless of commonalities in diet (Fig. 6a). Networks for Preb displayed a tendency for higher amounts of degree and significantly higher levels of stress at d 42 post-weaning (Fig. 6c and d), indicating that microbial communities were highly interconnected by the end of the nursery period. Although networks for treatment Preb changed in terms of which taxa were identified as prominent network members due to high degree or stress values from d 21 to 42 post-weaning, prominent taxa were all previously linked to fermentation and the production of short chain fatty acids (SCFAs) [22]. Unidentified species within the genus *Prevotella* were prominent network taxa for treatment Preb in terms of both network stress and degree, mirroring previous observations highlighting *Prevotella* as a keystone genus in nursery pig gut microbiome communities [3, 61], though accompanying increases in post-weaning growth rates were not observed for pigs in treatment Preb. However, it has also been proposed that nursery pig gut microbiome communities dominated by *Prevotella* in the immediate post-weaning period have initially slower weight gain that increases throughout the nursery period, ultimately conferring an advantage in compensatory growth rates due to increased ability to process complex dietary polysaccharides like those found in solid feed [3]. Interestingly, the taxa that were identified as having the highest degree for treatment Preb at d 42 post-weaning were all taxa that belonged to the discriminant genera identified for this treatment at d 21 post-weaning (Table 2). *Prevotella copri* was the most notable out of these taxa, as another prior network analysis involving stage-specific interactions between taxa observed that *Prevotella copri* served as a central node linking the transition in microbiome composition between the lactation and nursery stages [11]. These observations suggest that the discriminant genera identified for Preb at d 21 post-weaning were successfully integrated into the overall microbiome community structure and persisted throughout the nursery phase, even though they were not identified as discriminant genera at d 42 post-weaning. Overall, the construction of networks for each treatment group provides evidence that prebiotic supplementation with an *Aspergillus oryzae* fermentation extract is associated with transient stimulation of potentially beneficial fermentative bacteria, with potential implications for microbiome community function and performance.

### Interactions between dietary feed additives and maternal parity affect pig microbiome composition in the post-weaning period

The development and assembly of microbial communities is heavily influenced by the identity and relative diversity of the microbiome’s primary colonizers [11]. Primary colonizers have the ability to either promote or inhibit the growth of all other taxa that follow, through mechanisms of colonization resistance such as competitive exclusion [62, 63]. Therefore, differences in initial microbiome composition and colonization patterns driven by maternal parity have critical implications for how nursery pigs and their microbiomes respond to dietary interventions in the post-weaning period. Contrary to most studies in swine systems involving nutritional interventions in the post-weaning period, no significant interactions were observed between dietary treatment and maternal parity in growth performance models. However, significant interactions between dietary treatment and maternal parity were observed for nursery pig gut microbiome composition at d 42 post-weaning, though microbial composition was not significantly different among dietary treatments at this time-point (Fig. 4a and b). Gut microbiome composition of pigs receiving treatment Preb diverged according to maternal parity groups in terms of overall diversity (Fig. 5a–c) and composition (Fig. 5d–f, Additional files 7 and 8), with the effect size of these distinctions increasing throughout the nursery period (Fig. 5d–f). This observation directly contrasts with the overall observed trend of diminishing effect size for maternal parity throughout the nursery period (Fig. 3b), suggesting that prebiotic supplementation alone affects offspring of different maternal parity groups differently. In the absence of the antimicrobial properties associated with pharmacological supplementation of Zn and Cu [53], the growth of beneficial microorganisms was promoted in the gut microbiomes of pigs receiving the *Aspergillus oryzae* prebiotic immediately post-weaning (Table 2). However, the relative intensities of these fluctuations in abundance and their accompanying ramifications for microbiome community composition were likely driven by differences in initial community composition caused by maternal parity.

### Study limitations

Although minimal differences in sow gut microbiomes and no differences in sow vaginal microbiome compositions based on parity were observed in this study, these observations may be associated with the collection of samples at weaning rather than during gestation or shortly after parturition. This study was inherently constrained by the implementation of dietary treatments and phase-feeding in a manner that replicated standard nursery feeding regimens in commercial swine systems, meaning supplementation of dietary feed additives during only the first few weeks post-weaning potentially excluded further observations regarding their effects on microbiome composition at or beyond d 42 post-weaning. Therefore, we are unable to determine whether the lack of observed distinctions in microbial diversity and microbiome composition among treatment groups at d 42 post-weaning can be attributed to true biological patterns or the transition to a common diet between d 21 and d 42 post-weaning. Additionally, the use of a standard commercial nursery diet may have potentially introduced unknown antagonistic interactions between the feed additives. The use of 16S rRNA sequencing in this study mandates that all observations regarding potentially beneficial taxa lack the accompanying functional data to conclusively determine whether SCFA production is enhanced with prebiotic supplementation. Additionally, fecal consistency scoring to determine the potential beneficial health effects of prebiotic supplementation was not conducted.

## Conclusions

Our results indicate that the gut microbiomes of nursery pigs undergo structural development in a parity-dependent manner that can interact with dietary feed additives, specifically in the form of prebiotics or pharmacological doses of Zn and Cu. Nursery pig gut microbiome composition was influenced by supplementation with an *Aspergillus oryzae* fermentation extract, with varying results when combined with pharmacological levels of Zn and Cu or when comparing offspring belonging to different maternal parity groups. Future research regarding the relative effectiveness of dietary interventions in the post-weaning period should therefore be mindful of the potential for maternal parity to act as a confounding factor that could potentially exacerbate differences in the health and performance of primiparous pigs compared to multiparous pigs. Our results provide evidence that prebiotic supplementation alone in the immediate post-weaning period successfully promotes the growth and assimilation of beneficial microorganisms in the gut microbiomes of nursery pigs, though further investigation using metagenomics and metabolomics techniques to gather accompanying functional data is necessary. Future studies involving the timing and duration of prebiotic supplementation in nursery pig diets, as well as following pigs longitudinally to market, are necessary to conclusively determine whether prebiotic supplementation with an *Aspergillus oryzae* fermentation extract creates long-lasting effects on the microbiome and performance of pigs.

### Supplementary Information

**Additional file 1.** Ingredient composition and calculated energy and nutrient content of diets. Diets were fed in four phases, and represent the base diets prior to additions of pharmacological doses of Zinc/Copper or Prebiotic according to treatment groups.

**Additional file 2.** Summary statistics for sequencing reads. Copy numbers from qPCR and numbers of reads prior to and after sequence data processing display adequate coverage across all samples. Percentage of barcodes, percentage of one mismatch bases, yield, percentage of reads over a mean quality score of 30, and mean quality for each sample are listed.

**Additional file 3.** Sow reproductive performance. Average preweaning mortalities and number of piglets liveborn are listed for each maternal parity group, as well as for all sows in the study combined. Data is presented as average counts with standard deviation, with significant differences denoted with differing letter superscripts (*t*-test, *P* < 0.05).

**Additional file 4.** Effects of dietary treatment group on nursery pig ADG and ADFI. Average overall nursery ADG and ADFI are listed for each treatment, as well as averages of weekly ADFI. ADG was calculated by dividing total nursery weight gain by the number of d in the study (42 d). ADFI was calculated on a weekly basis by dividing feed disappearance by the number of pigs in each pen. Pooled standard error for each metric is also listed. Significant differences among dietary treatment groups are denoted with differing letter superscripts (*P*<0.05).

**Additional file 5.** Discriminant taxa between parity groups for sow vaginal microbiomes at weaning (d 0). Listed taxa were selected on the basis of having indicator value scores of at least 0.5, and significant differences (Wilcoxon test, *P *< 0.05) in average relative abundances between parity groups. Group designations of P or M represent the parity group (Primiparous or Multiparous) the listed taxa is enriched in. *P*-values represent One-tailed Wilcoxon tests between parity groups. Listed taxa are identified to the genus (g) level, or represent unidentified genera within the listed family (f), order (o), class (c), or phylum (p), where applicable.

**Additional file 6.** Discriminant taxa between parity groups for sow gut microbiomes at weaning (d 0). Listed taxa were selected on the basis of having indicator value scores of at least 0.5, and significant differences (Wilcoxon test, *P* < 0.05) in average relative abundances between parity groups. Group designations of P or M represent the parity group (Primiparous or Multiparous) the listed taxa is enriched in. Taxa are sorted by parity group and then by indicator value. *P*-values represent One-tailed Wilcoxon tests between parity groups. Listed taxa are identified to the genus (g) level, or represent unidentified genera within the listed family (f), order (o), class (c), or phylum (p), where applicable.

**Additional file 7.** Discriminant taxa among dietary treatment groups for primiparous offspring gut microbiomes. Listed taxa were selected on the basis of having indicator value scores of at least 0.5, and significant differences (Kruskal Wallis, *P* < 0.05) in average relative abundances between parity groups. Treatment designations represent the dietary treatment group the listed taxa is enriched in. If a group is not listed, no discriminant taxa with the listed criteria were observed. Taxa are sorted by treatment group and then by indicator value for each time-point. *P*-values represent Kruskal Wallis tests among treatment groups. Listed taxa are identified to the genus (g) level, or represent unidentified genera within the listed family (f), order (o), class (c), or phylum (p), where applicable.

**Additional file 8.** Discriminant taxa among dietary treatment groups for multiparous offspring gut microbiomes. Listed taxa were selected on the basis of having indicator value scores of at least 0.5, and significant differences (Kruskal Wallis, *P* < 0.05) in average relative abundances between parity groups. Treatment designations represent the dietary treatment group the listed taxa is enriched in. If a group is not listed, no discriminant taxa with the listed criteria were observed. Taxa are sorted by treatment group and then by indicator value for each time-point. *P*-values represent Kruskal Wallis tests among treatment groups. Listed taxa are identified to the genus (g) level, or represent unidentified genera within the listed family (f), order (o), class (c), or phylum (p), where applicable.

## Data Availability

The datasets used in the current study are available from the corresponding author on reasonable request.

## Abbreviations

ADFI: Average daily feed intake
ADG: Average daily gain
ASPL: Average shortest path length
Con: Control dietary treatment group, including Zinc and Copper
Cu: Copper
DFM: Direct fed microbial
Preb: Dietary treatment group supplemented with *Aspergillus oryzae* prebiotic
Preb + ZnCu: Dietary treatment group supplemented with the *Aspergillus oryzae* prebiotic, Zinc, and Copper
SCFA: Short chain fatty acid
Zn: Zinc
